# Editorial: New insights in molecular pathways in ototoxicity

**DOI:** 10.3389/fncel.2023.1202490

**Published:** 2023-05-05

**Authors:** Qianqian Yang, Chen Chen, Jianfeng Li

**Affiliations:** ^1^Department of Pathology, The First Affiliated Hospital of Soochow University, Suzhou, Jiangsu, China; ^2^Department of Otolaryngology — Head and Neck Surgery, Stanford University School of Medicine, Stanford, CA, United States; ^3^Stanford Cancer Institute, Stanford University School of Medicine, Stanford, CA, United States; ^4^Department of Otolaryngology — Head and Neck Surgery, Shandong Provincial Hospital, Shandong University, Jinan, Shandong, China; ^5^Institute of Eye and ENT, Provincial Hospital Affiliated to Shandong First Medical University, Jinan, Shandong, China

**Keywords:** cisplatin, mitophagy, regeneration, development, Ménière's disease

Ototoxicity refers to damage to the inner ear, characterized by cochlear (cochleotoxicity) or vestibular (vestibulotoxicity) dysfunction (Steyger et al., 2018). Auditory dysfunction is one of the major global health issues, affecting ~466 million people worldwide, and can be categorized into conductive and sensorineural hearing losses (SNHL) (Zhang L. et al., 2022). SNHL is primarily caused by intrinsic and extrinsic damage to hair cells (HCs) and spiral ganglion neurons (SGNs). These cells have a limited ability to repair and regenerate during maturation, particularly in mammals, resulting in permanent hearing loss.

This Research Topic, “*New insights in molecular pathways in ototoxicity*,” includes original research articles and focused reviews on the mechanisms underlying inner ear damage, as well as the development and regeneration of HCs, which may provide a potential pathway to repair and restore hearing.

According to the systematic review by Han et al. included in this Research Topic, ototoxic factors damage HCs mainly by inducing mitochondrial dysfunction. Mitochondria are responsible for energy production and play a key role in maintaining cellular homeostasis and facilitating cellular metabolic events. In ototoxicity, mitochondrial dysfunction is linked to the generation of free radicals, increases in free Ca^2+^, mitochondrial DNA mutations, and mitochondrial apoptosis. Mitophagy, the selective degradation of mitochondria, is a conserved mechanism that regulates cell quality and homeostasis, from yeast to humans. Mitophagy has also been implicated in regulating the occurrence and progression of ototoxicity. Several ototoxicity models have demonstrated disrupted mitophagy, including drug-induced, noise-induced, and age-related deafness. Mitophagy has been identified as a potential target for therapeutic intervention in ototoxicity due to its ubiquity and role in the disease. Certain compounds or drugs, such as ginseng and Mdivi-1, have been shown to alleviate ototoxicity by targeting mitophagy. However, the involvement of mitophagy in ototoxicity is complex and dynamic, and further research is needed to clarify the process and develop practical applications.

Cisplatin is a widely used chemotherapy agent for the treatment of various solid tumors. However, its clinical utility is limited due to the ototoxicity it induces. Although the mechanism underlying cisplatin-related ototoxicity has been studied for more than half a century (Fleischman et al., 1975), the details have not been fully elucidated. Among the different cytotoxic outcomes, inflammation plays a critical role in cochlear dysfunction, mediated by various inflammatory cytokines and immune cells, including resident cells and migrated cells (Keithley, 2022). Recent research by Al Aameri et al. has implicated the chemokine CXCL1 as an early mediator of cisplatin ototoxicity. By initiating an immune cascade through binding to its receptor, CXCR2, CXCL1 can lead to cochlear inflammation, pathology, and hearing loss. During this process, various cells in the cochlea, including spiral ganglion cells, outer hair cells, and Deiters' cells, can serve as sources of the chemokine CXCL1, which increases immunoreactivity. Inhibition of this pathway reduces immune cell migration and mitigates cisplatin-induced synaptopathy and hearing loss, which may serve as a promising treatment for cisplatin-induced hearing loss.

SNHL is irreversible, making hearing restoration a challenging issue. To make breakthroughs in hearing restoration, a deep understanding of inner ear development is crucial. Recent research by Liu et al. sheds light on the development of hair cells in zebrafish, which could help us understand this process more thoroughly. Liu et al. first examined the expression of LOXHD1B, a mutation that causes high- and intermediate-frequency hearing loss in patients, in HEI-OC1 cells, the C57BL/6 mouse cochlea and inner ear, and zebrafish olfactory pores. They found a reduced number of spinal and lateral line neuromasts in the inner ear of Loxhd1b knockdown zebrafish, accompanied by impaired hearing, which confirms the function of Loxhd1b in zebrafish hair cell development and hearing. Transcriptome sequencing revealed that brain-derived neurotrophic factor (BDNF) is a downstream molecule of LOXHD1B, while LOXHD1B and BDNF regulate zebrafish hair cell formation through synergistic regulation of the TrkB/ERK pathway. This study identifies BDNF as a key molecule for further exploration in the treatment of hereditary deafness.

Studies have shown that immature supporting cells (SCs) in neonatal mice have limited capacity for HC regeneration before the onset of hearing (Li et al., 2022). HCs can regenerate through mitotic division followed by differentiation by activating WNT signaling (Chai et al., 2012) or directly transdifferentiating by blocking NOTCH signaling (McGovern et al., 2018), neither of which are functional during maturation (Brown and Groves, 2020). However, it is still uncertain whether quiescent adult mammalian cochlear progenitors exist (Hoa et al., 2020), and whether their potential can be activated by a specific microenvironment. Birds have robust sensory hair cell regeneration at any age, during which the epidermal growth factor receptor (EGFR) family is required for the proliferation of inner ear-supporting cells (White et al., 2012). ERBB2 is a receptor tyrosine kinase in the EGFR family that can preferentially heterodimerize with the other three EGFR family members (ERBB1, ERBB3, and ERBB4) and drives proliferation of SCs in explant cultures and supernumerary HC-like formations in the neonatal mouse cochlea (Zhang et al., 2018). Piekna-Przybylska et al. performed single-cell RNA sequencing (scRNA-seq) on neonatal SCs with and without CA-ERBB2, a mutant ErbB2 transgene encoding a CA-ERBB2 protein, to identify ERBB2-mediated signaling pathways. The results showed that ERBB2 induction *in vivo* generated a new population of cells with *de novo* expression of a gene network whose ligands and regulators alter NOTCH signaling and promote cell survival, proliferation, and differentiation in other systems. The proliferating cochlear cells aggregate in the organ of Corti. The results also suggest that ectopic activation of ERBB2 signaling in cochlear SCs can alter the microenvironment to promote proliferation and cell rearrangements, which may represent a novel mechanism for inducing stem cell-like activity in the adult mammalian cochlea.

When discussing vestibular dysfunction, Ménière's disease (MD) must be mentioned, as it is primarily characterized by endolymphatic hydrops (EH) in the cochlear duct and vestibular labyrinth, leading to symptoms such as ear fullness, tinnitus, fluctuating sensorineural hearing loss, and vertigo (Oguz et al., 2021). The current clinical treatment approach for MD focuses primarily on symptom relief due to a lack of understanding of the underlying mechanisms of this condition. Recent studies have indicated that inflammation is an important pathogenic factor in MD, although the molecular mechanisms remain unclear (Zhang N. et al., 2022). Zhang et al. discovered that the interleukin (IL)-1 signaling pathway plays a key role in lipopolysaccharide (LPS)-induced EH in a mouse model. Furthermore, anakinra, a recombinant, non-glycosylated form of human IL-1RA, could interfere with the binding of IL-1 to its receptor, thereby reducing EH, improving auditory and vestibular function, and protecting the cochlear nerve. The evidence presented in this paper demonstrates that inhibiting the IL-1 signal pathway is a promising approach for the treatment of MD.

In summary, the Research Topic comprises five papers in the field of ototoxicity. This collection of studies presents some new ideas and findings for understanding the mechanisms of ototoxicity. We hope that scientists around the world will be inspired by the topics discussed here.

## Author contributions

QY wrote the original draft. CC revised the editorial. All authors revised and approved this editorial.
